# Bottled water contaminant exposures and potential human effects

**DOI:** 10.1016/j.envint.2022.107701

**Published:** 2022-12-15

**Authors:** Paul M. Bradley, Kristin M. Romanok, Kelly L. Smalling, Michael J. Focazio, Nicola Evans, Suzanne C. Fitzpatrick, Carrie E. Givens, Stephanie E. Gordon, James L. Gray, Emily M. Green, Dale W. Griffin, Michelle L. Hladik, Leslie K. Kanagy, John T. Lisle, Keith A. Loftin, R. Blaine McCleskey, Elizabeth K. Medlock–Kakaley, Ana Navas-Acien, David A. Roth, Paul South, Christopher P. Weis

**Affiliations:** a U.S. Geological Survey, Columbia, SC, USA; b U.S. Geological Survey, Lawrenceville, NJ, USA; c U.S. Geological Survey, Reston, VA, USA; d U.S. Environmental Protection Agency, Durham, NC, USA; e U.S. Food and Drug Administration, College Park, Maryland, USA; f U.S. Geological Survey, Lansing, MI, USA; g U.S. Geological Survey, Kearneysville, WV, USA; h U.S. Geological Survey, Lakewood, CO, USA; i U.S. Geological Survey, Saint Petersburg, Florida, USA; j U.S. Geological Survey, Sacramento, CA, USA; k U.S. Geological Survey, Lawrence, KS, USA; l U.S. Geological Survey, Boulder, CO, USA; m Columbia University, New York, New York, USA; n National Institute of Environmental Health Sciences/NIH, Bethesda, MD, USA

**Keywords:** Bottled water, Contaminant mixtures, Organics, Inorganics, Microorganisms, Human health

## Abstract

**Background:**

Bottled water (BW) consumption in the United States and globally has increased amidst heightened concern about environmental contaminant exposures and health risks in drinking water supplies, despite a paucity of directly comparable, environmentally-relevant contaminant exposure data for BW. This study provides insight into exposures and cumulative risks to human health from inorganic/organic/microbial contaminants in BW.

**Methods:**

BW from 30 total domestic US (23) and imported (7) sources, including purified tapwater (7) and spring water (23), were analyzed for 3 field parameters, 53 inorganics, 465 organics, 14 microbial metrics, and *in vitro* estrogen receptor (ER) bioactivity. Health-benchmark-weighted cumulative hazard indices and ratios of organic-contaminant *in vitro* exposure-activity cutoffs were assessed for detected regulated and unregulated inorganic and organic contaminants.

**Results:**

48 inorganics and 45 organics were detected in sampled BW. No enforceable chemical quality standards were exceeded, but several inorganic and organic contaminants with maximum contaminant level goal(s) (MCLG) of zero (no known safe level of exposure to vulnerable sub-populations) were detected. Among these, arsenic, lead, and uranium were detected in 67 %, 17 %, and 57 % of BW, respectively, almost exclusively in spring-sourced samples not treated by advanced filtration. Organic MCLG exceedances included frequent detections of disinfection byproducts (DBP) in tapwater-sourced BW and sporadic detections of DBP and volatile organic chemicals in BW sourced from tapwater and springs. Precautionary health-based screening levels were exceeded frequently and attributed primarily to DBP in tapwater-sourced BW and co-occurring inorganic and organic contaminants in spring-sourced BW.

**Conclusion:**

The results indicate that simultaneous exposures to multiple drinking-water contaminants of potential human-health concern are common in BW. Improved understandings of human exposures based on more environmentally realistic and directly comparable point-of-use exposure characterizations, like this BW study, are essential to public health because drinking water is a biological necessity and, consequently, a high-vulnerability vector for human contaminant exposures.

## 1. Introduction

The quality and long-term sustainability of drinking water (drinking/cooking water, collectively) are societal priorities and increasing concerns in the United States (US) (Allaire et al. 2018; Javidi and Pierce 2018; Pierce and Gonzalez 2017) and worldwide (Doria, 2010; Tröger et al. 2018; Villanueva et al. 2014), due to, among other reasons, population-driven increases in water use/reuse demands (DeSimone et al. 2015; Dieter et al. 2018) and in anthropogenic source-water contamination (Bexfield et al. 2019; Bexfield et al. 2021; DeSimone et al. 2015; Toccalino et al. 2012). In the US and globally, drinking water is delivered to consumers via three general supply chains or distribution “pipelines” (public tapwater [TW], private TW, bottled water [BW]), each with distinct logistical, infrastructure, regulatory, and commercial profiles, but all similarly challenged by an increasingly anthropized water cycle. Many water-borne pathogens and contaminants are actively regulated and monitored in US public-supply TW under the Safe Drinking Water Act (SDWA) (U.S. Environmental Protection Agency 2021a, e) and in BW as a food under the Food, Drugs, and Cosmetics Act (FD&C Act) (U.S. Food & Drug Administration 2021) and corresponding amendments; private–supply TW, however, is not systematically regulated or monitored (U.S. Environmental Protection Agency 2021b). Despite these regulatory differences, anthropogenic (i.e., human-–generated or –driven) contaminant concerns are common to all three pipelines because existing drinking-water regulations (Health Canada 2020; U.S. Environmental Protection Agency 2021a,e; World Health Organization (WHO) 2011) do not encompass many of the anthropogenic chemicals reported in ambient surface-water or groundwater source waters (Bradley et al. 2017; de Jesus Gaffney et al. 2015; DeSimone et al. 2015; Toccalino et al. 2012), much less the hundreds of thousands of synthetic chemicals estimated to be in commercial use globally (Wang et al. 2020).

The United States Geological Survey (USGS) collaborates with the Environmental Protection Agency (EPA), Food and Drug Administration (FDA), National Cancer Institute (NCI), National Institute of Allergy and Infectious Disease (NIAID), National Institute of Environmental Health Science (NIEHS), tribal nations, universities, water utilities, communities, and others to inform drinking-water exposure and water-supply data gaps by assessing inorganic/organic/microbial contaminant mixtures in point–of–use (POU) drinking water (Bradley et al. 2018; Bradley et al. 2020; Bradley et al. 2021a; Bradley et al. 2021b). Sampling personnel, collection protocols, core target-analytical methods and laboratories, and quality assurance/quality control procedures are maintained to ensure direct comparability across study areas and drinking-water distribution pipelines. Studies to date have focused on assessing contaminant mixtures in private– and public–supply TW and their associated distal (e.g., ambient source water) and proximal (e.g., premise plumbing, POU treatment) drivers in a range of socioeconomic and source-water vulnerability settings across the US. In 2020, USGS, FDA, EPA, and NIEHS conducted a reconnaissance of simultaneous inorganic/organic/microbial exposures in a cross–section (30 total) of individual-serving BW available in the US. This study was initiated to provide insight into cumulative contaminant risk (Moretto et al. 2017; National Research Council 1983; Norton et al. 1992) to human health from contaminants in BW and to expand the national perspective on inorganic/organic/microbial contaminant exposures in POU drinking-water by maintaining the approach employed across the US in previous POU TW studies by this group (Bradley et al. 2018; Bradley et al. 2020; Bradley et al. 2021a; Bradley et al. 2021b; Bradley et al. 2022).

For this study, BW exposure was operationally represented as detections (concentrations) of 53 inorganic and 465 unique organic analytes, 14 microbial metrics, and 1 *in vitro* bioactivity in BW samples. Potential human-health risks of individual and aggregate TW exposures were explored based on two lines of evidence: 1) cumulative detections and concentrations of designed-bioactive (e.g., pesticides, pharmaceuticals) chemicals (Bradley et al., 2017; Bradley et al. 2018; Bradley et al. 2020) and 2) exposure metrics based on cumulative Exposure-Activity Ratio(s) (Σ_*EAR*_) (Blackwell et al. 2017) and hazard indices (HI, cumulative toxicity/hazard quotients for mixtures (Goumenou and Tsatsakis 2019; U.S. Environmental Protection Agency 2011, 2012)) of cumulative benchmark-based Toxicity Quotients (Σ_TQ_) (Corsi et al. 2019).

Multiple TW-exposure hypotheses, relevant to BW specifically and to POU drinking water in general, were assessed. In line with an increasingly anthropized water cycle and with previous TW results by this research group (Bradley et al. 2018; Bradley et al. 2020; Bradley et al. 2021a; Bradley et al. 2021b; Bradley et al. 2022) and others (e.g., de Jesus Gaffney et al. 2015; Focazio et al. 2006; Knobeloch et al. 2013; Postma et al. 2011; Rogan and Brady 2009), simultaneous exposures to multiple inorganic and organic constituents of potential human-health interest were expected to occur in BW samples (Hypothesis I). Exceedances of FDA-enforceable BW standard of quality (SOQ, “shall not contain in excess of”) levels (U.S. Food & Drug Administration 2021); adopted from and, with few exceptions (e.g., lead [Pb]), equivalent to EPA public-supply enforceable National Primary Drinking Water Regulation maximum contaminant level(s) (MCL) (U.S. Environmental Protection Agency 2021a, e); were not expected (Hypothesis II). However, exceedances of EPA MCL goal(s) (MCLG, maximum level of a contaminant in drinking water at which no known or anticipated adverse effect on the health of sensitive subpopulations would occur, allowing an adequate margin of safety) (U.S. Environmental Protection Agency 2021d), other non-enforceable health-only advisories, or stricter state-promulgated enforceable standards were expected to occur in some BW samples (Hypothesis III).

## 2. Methods

### 2.1. Source selection and sampling

For this reconnaissance of potential human exposures to an expanded range of inorganic, organic, and microbial contaminants in BW, a cross-section of 30 BW brands (anonymized) available commercially in the US were selected to cover a variety of 1) source locations (US domestic, imported), 2) source types (spring or artesian [referred to collectively as “spring”], “purified” public-supply TW [purified-TW]), 3) purification treatments, and 4) packaging materials (glass, aluminum, carton) (Table S1) and analyzed one time each. For organic–chemical analyses and bioassays, samples were prepared by pouring water from the original BW packaging into the appropriate analytical sample bottle at the USGS New Jersey Water Science Center laboratory and were then shipped on ice overnight to the respective analytical laboratory. Controls for sampling artifacts (nominal field blanks) were prepared in the same manner and location using reagent blank waters. For inorganic–chemical and microbial analyses, BW samples were delivered in their original packaging to the analytical laboratory for processing and analysis.

### 2.2. Analytical methods

Briefly, BW samples were analyzed by USGS using 5 inorganic (53 analytes), 8 target-organic (465 unique analytes), 3 field parameter, and 14 microbial methods (Table S2), as discussed (Bradley et al. 2018; Bradley et al. 2020; Bradley et al. 2021a; Romanok et al. 2018) and described in detail previously (American Public Health Association 2018a,b,c,d; Ball and McCleskey 2003; Barringer and Johnsson 1996; Cohn et al. 1968; Furlong et al. 2014; Graham et al. 2010; Hajna 1955; Hergenreder 2011; Hladik et al. 2014; Hoffman et al. 1996; Kolpin et al. 2021; Kozak et al. 2013; Levin and Cabelli 1972; Lilley and Brewer 1953; Lisle and Priscu 2004; Loftin et al. 2016; McCleskey et al. 2019; Petrisek and Hall 2017; Rose et al. 2016; Sandstrom et al., 2015; U.S. Environmental Protection Agency 1997, 2014; U.S. Geological Survey variously dated). Pharmaceutical and pesticide samples were syringe filtered (0.7 μm nominal pore size, glass fiber) prior to analysis (Furlong et al. 2014; Sandstrom et al., 2015). The T47D-KBluc (American Type Cell Culture, ATCC, Manassas, VA; #CRL-2865) estrogen receptor transcriptional activation bioassay, previously developed (Wilson et al. 2004) and applied to environmental samples (Conley et al. 2017a; Medlock Kakaley et al. 2020) and treated tapwater (Conley et al. 2017b; Medlock Kakaley et al. 2021) was used to screen bottled water extracts for estrogenic activity (Neale et al. 2021). Cell culture maintenance and bioassay were performed as previously described (Wilson et al. 2004) with exceptions (Medlock Kakaley et al. 2020). Cells were exposed to a 17β-estradiol (E2; CAS #: 50-28-2; purity 98 %; catalog no. E887; lot: 28H0818) standard curve (0, 0.3, 1.0, 3.0, 10, and 30 pM), ICI 182,780 (CAS #: 72795-01-8; purity 99 %; catalog no. 1047) antagonist control, methanol control, or bottled water extract. Extracts were resuspended in methanol, diluted in bioassay media, and screened at 5 and 10 times the final concentration of the original water sample. Each sample was dosed in quadruplicate and analyzed across at least 3 replicate 96-well plates (i.e., unique cell passage). Luminescence (relative light units; RLU) was measured using ClarioStar luminometer (BMG LabTech, Cary, NC). Data and statistical analysis were performed using GraphPad Prism 8.0 (GraphPad Software, La Jolla, California) and SAS statistical software (Cary, NC USA), as previously described (Medlock Kakaley et al. 2021). All results are in Tables S3–S5 and in Romanok et al. (2022).

### 2.3. Quality assurance

Quantitative (≥limit of quantitation, ≥LOQ) and semi-quantitative (between LOQ and long-term method detection limit, MDL (Childress et al. 1999; U.S. Environmental Protection Agency 2020b)) results were treated as detections (Childress et al. 1999; Foreman et al. 2021; Mueller et al. 2015). Chemical quality-assurance/quality-control included 3 nominal field blanks (organics, inorganics) as well as laboratory blanks (organics, inorganics), spikes (organics), and stable–isotope surrogates (organics) prepared at respective analytical laboratories. The median organic surrogate recovery (Table S4c) was 98.5 % (interquartile range: 88.5–108 %). Despite infrequent detections and very low detected concentrations in inorganic blanks, maximum blank concentrations for bromide, sulfate, and zinc were nevertheless within the range observed in some BW samples (Table S3c); corresponding results were censored at the analyte-specific maximum blank concentration, as footnoted (Table S3a). Only chlorodifluoromethane (HCFC-22; 0.02 μg L^−1^), 1,1-difluoroethane (0.01 μg L^−1^), ethyl acetate (0.09 μg L^−1^), and n-pentanal (0.011 μg L^−1^) were detected in blanks (once each) at concentrations in the range observed in BW samples (Table S4b); corresponding results were censored at twice the analyte-specific maximum blank concentration and, consequently, HCFC-22 was removed from the interpretive dataset, as footnoted (Table S4a). No growth was detected for any microbial quality-assurance/quality-control sterile laboratory blank, as footnoted (Tables S5).

### 2.4. Statistics

Differences (centroids and dispersions) between BW-sample groups were assessed by nonparametric One–way PERMANOVA (n = 9999 permutations) on Euclidean distance (Paleontological Statistics, PAST, vers. 4.03) (Hammer et al. 2001). Relations between detected BW contaminants were assessed by Spearman rank-order (rho (ρ)) correlation and permuted (n = 9999 permutations) probabilities (PAST, vers. 4.03) (Hammer et al. 2001).

### 2.5. Risk assessments

A screening-level assessment (Goumenou and Tsatsakis 2019; U.S. Environmental Protection Agency 2011) of potential cumulative biological activity of mixed-organic contaminants in each BW sample was conducted as described (Blackwell et al. 2017; Bradley et al. 2018; Bradley et al., 2019). The toxEval version 1.2.0 package (De Cicco et al. 2018) of the open source statistical software R (R Development Core Team 2019) was used to sum (non-interactive concentration addition model (e.g., Altenburger et al. 2018; Cedergreen et al. 2008; Stalter et al. 2020) individual EAR (ratio of the detected concentration to the activity concentration at cutoff (ACC) from the Toxicity ForeCaster (U.S. Environmental Protection Agency 2020a; U.S. Environmental Protection Agency National Center for Computational Toxicology 2020) high-throughput screening data (U.S. Environmental Protection Agency National Center for Computational Toxicology 2020)) to estimate sample-specific cumulative EAR (Σ_EAR_) (Blackwell et al. 2017; Bradley et al. 2018; Bradley et al. 2020). ACC estimates the point of departure concentration at which a defined threshold of response (cutoff) is achieved for a given biological activity and is less prone to violations of relative potency assumptions (for discussion see, Blackwell et al. 2017). ACC data in the toxEval v1.2.0 employed in the present study were from the August 2020 invitroDBv3.2 release of the ToxCast database (U.S. Environmental Protection Agency 2020a; U.S. Environmental Protection Agency National Center for Computational Toxicology 2020). Non-specific-endpoint, baseline, and unreliable response-curve assays were excluded (Blackwell et al. 2017; Bradley et al. 2021a; Bradley et al. 2021b). Σ_EAR_ results and exclusions are summarized in Tables S6a–6b.

An analogous human-health-benchmark HI assessment (Goumenou and Tsatsakis 2019; U.S. Environmental Protection Agency 2011, 2012) of the combined inorganic and organic contaminant risk also was conducted using toxEval v1.2.0 (De Cicco et al. 2018) to sum the toxicity quotient (TQ, ratio of detected concentration to corresponding health–based benchmark) of individual detections to estimate sample-specific cumulative TQ (Σ_TQ_) (Corsi et al. 2019). A precautionary screening–level approach was employed based on the most protective human–health benchmark (i.e., lowest benchmark concentration) among maximum contaminant level (MCL) goal (MCLG) (U.S. Environmental Protection Agency 2017, 2021e), World Health Organization (WHO) Guideline Values (GV) and provisional GV (pGV) (World Health Organization (WHO) 2011), USGS Health-Based Screening Level (HBSL; (Norman et al. 2018)), and state drinking-water MCL or drinking-water health advisories (DWHA). For the Σ_TQ_ assessment, MCLG values of zero (i.e., no identified safe-exposure level for sensitive sub-populations, including infants, children, the elderly, and those with compromised immune systems and chronic diseases (U.S. Environmental Protection Agency 2021d, e)) were set to the respective method reporting limit, except for Pb, which was set to 1 μg L^−1^ as suggested by the American Academy of Pediatrics (Lanphear et al. 2016). Σ_TQ_ results and respective health–based benchmarks are summarized in Tables 7a–7b. Corsi et al. (2019) reported approximate contaminant-specific equivalency of the widely employed TQ = 0.1 screening-level threshold of concern and EAR = 0.001.

## 3. Results and discussion

**Fig. 1.**
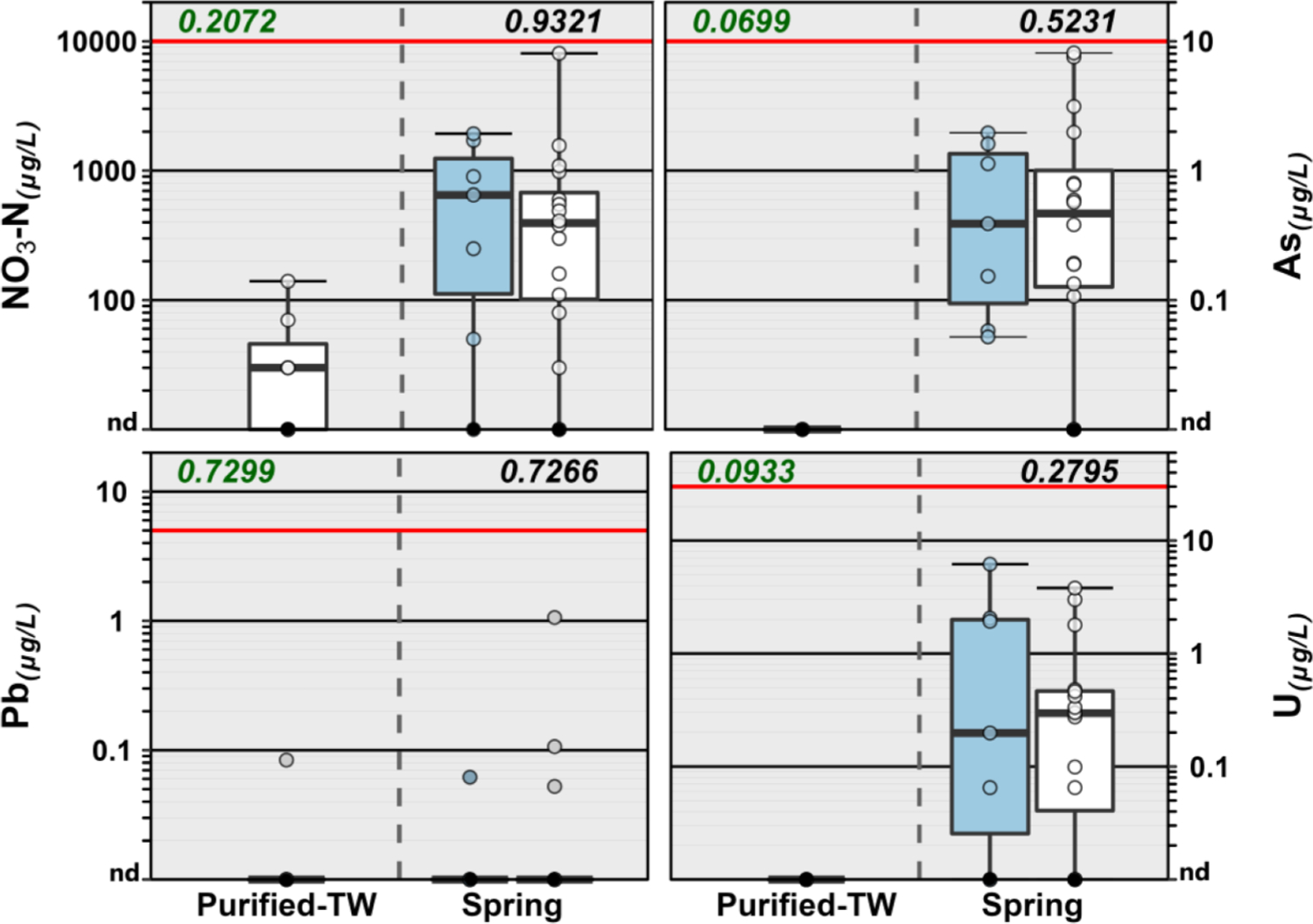
Group comparison of concentrations of select inorganics detected in purified-TW (domestic) and spring (domestic, white; imported, blue) sourced bottled water samples during 2020. Solid red lines indicate enforceable FDA SOQ levels. EPA MCLG for As, U, and Pb are zero. For NO_3_-N, SOQ and MCLG are the same. Boxes, centerlines, and whiskers indicate interquartile range, median, and 5th and 95th percentiles, respectively. Numbers in green font (top left of plots) indicate the permuted probability that the centroids and dispersions are the same (PERMANOVA; 9999 permutations) across all (purified-TW and spring sourced) BW groups; numbers above spring-sourced BW boxplot pairs (top right of plots) indicate the permuted probability that the centroids and dispersions are the same for spring-sourced BW groups. “nd” indicates not detected.

**Fig. 2.**
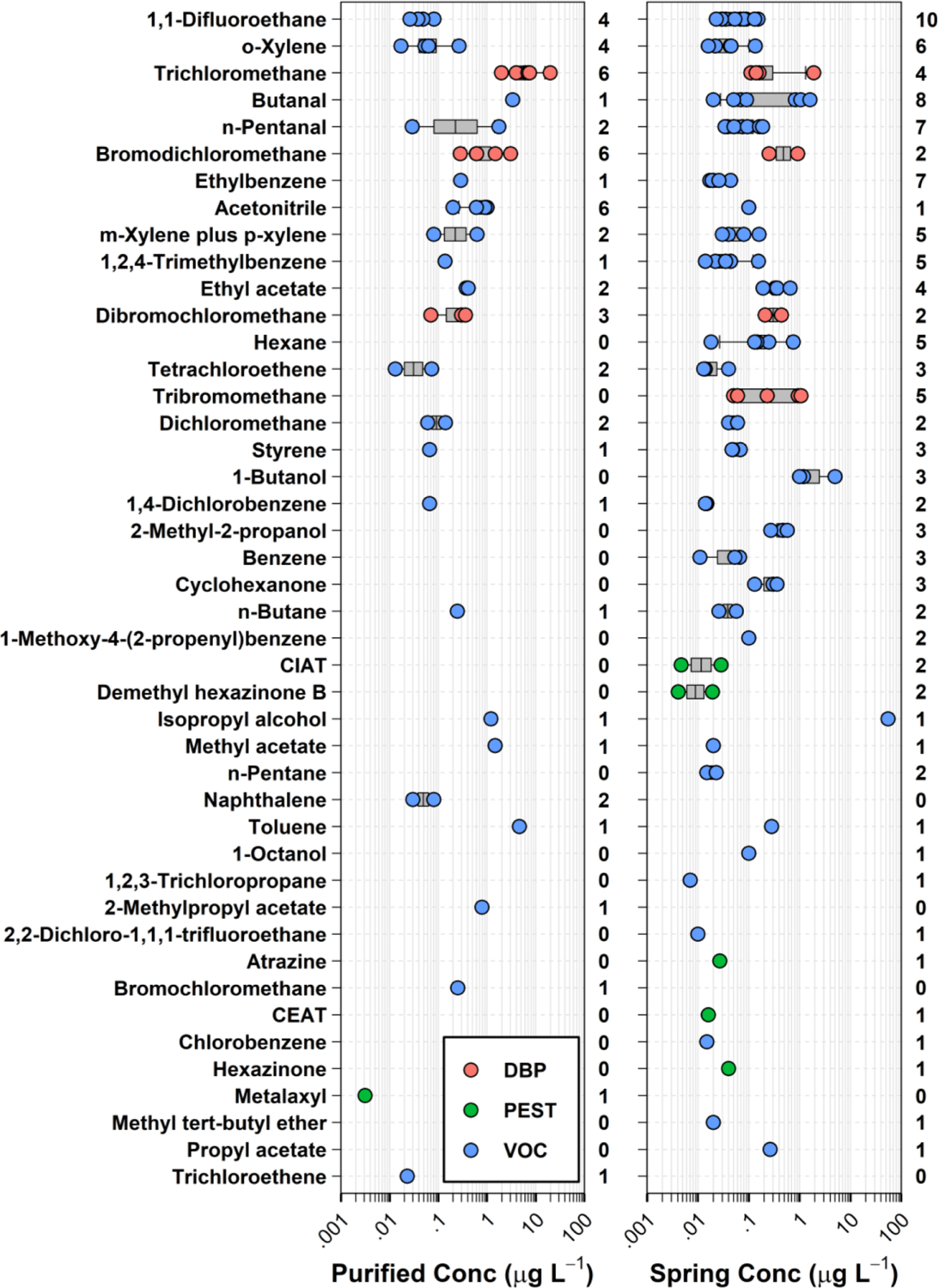
Detected concentrations (circles, μg L^−1^) and number of bottled water samples (right axes) for 45 organic analytes (left axis, in order of decreasing total detections) detected in purified-TW (left plot) and spring (right plot) sourced bottled water samples during 2020. Circles (●) are data for individual samples. Boxes, centerlines, and whiskers indicate interquartile range, median, and 5th and 95th percentiles, respectively. DBP, PEST, and VOC indicate disinfection byproducts, pesticides, and volatile organic chemicals, respectively. VOC generally associated with disinfection processes when present in drinking water are identified as DBP. CEAT and CIAT are deisopropylatrazine and deethylatrazine, respectively.

**Fig. 3.**
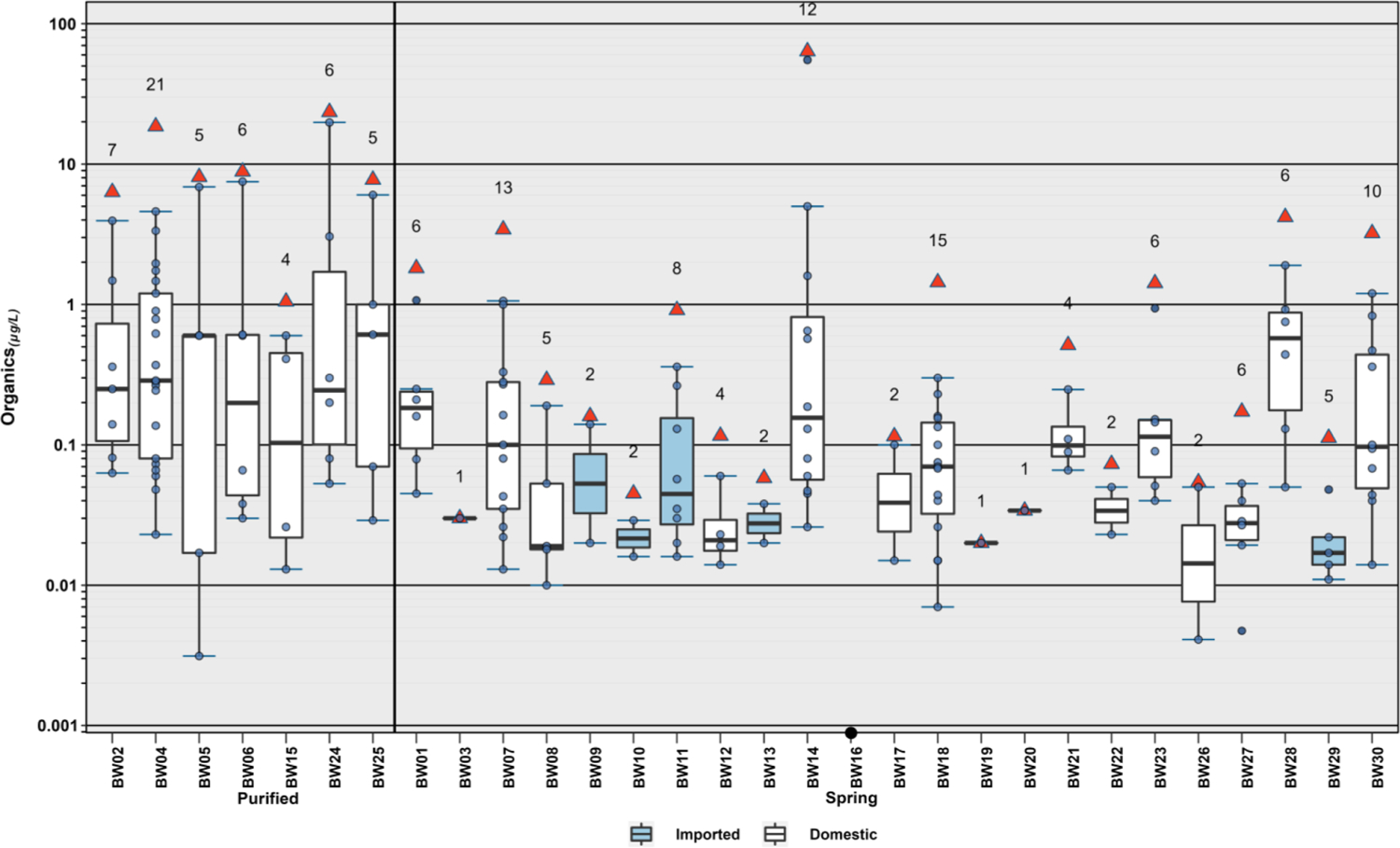
Individual (circles, ●) and cumulative (sum of all detected; red triangles, △) concentrations of 45 organic analytes detected in spring (domestic, white; imported, blue) and purified-TW (domestic, white) sourced bottled water samples during 2020. Boxes, centerlines, and whiskers indicate interquartile range, median, and 5th and 95th percentiles, respectively. Numbers above each boxplot indicate total detected organic analytes. Circle on x-axis (BW16) indicates no organic analytes detected.

**Fig. 4.**
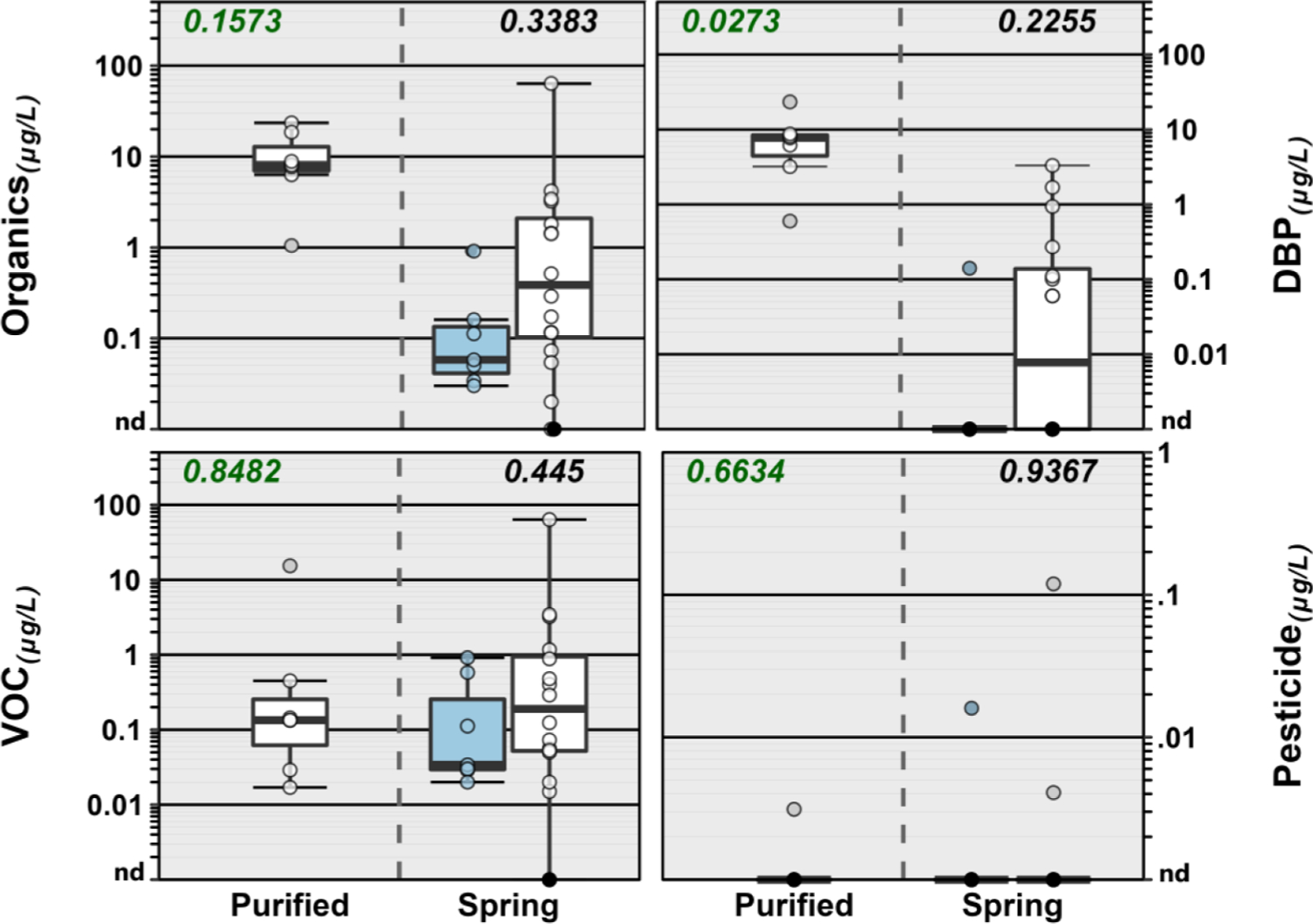
Group comparisons of cumulative concentration of all organics (upper left plot) and select organic classes detected in spring (domestic, white; imported, blue) and purified-TW (domestic) sourced bottled water samples during 2020. Boxes, centerlines, and whiskers indicate interquartile range, median, and 5th and 95th percentiles, respectively. Numbers in green font (top left of plots) indicate the permuted probability that the centroids and dispersions are the same (PERMANOVA; 9999 permutations) across all (purified-TW and spring sourced) BW groups; numbers above spring-sourced BW boxplot pairs (top right of plots) indicate the permuted probability that the centroids and dispersions are the same for spring-sourced BW groups. “nd” indicates not detected.

Consistent with an increasingly anthropized water cycle and with the results of previous POU–TW studies by this group, regulated and unregulated chemical (inorganic, organic) and microbial analytes were routinely detected in BW samples (Tables S3–S5; Figures 1–4, S1), with 2 or more detections of potential human–health concern often observed per sample. Approximately 91 % (48) of the 53 inorganic analytes and 10 % (45) of the 465 unique organic–indicator analytes were detected at least once in BW. Bacteria were broadly detected in BW by direct counts (83 % of samples) and by growth on non-selective heterotrophic plate media (70 %), with detection of growth on at least one putative pathogen selective media in 24 (80 %) of the tested BW samples.

Organ/organism–level human–health effects are screened herein based on MCLG and other human-health drinking-water advisories that define a margin-of-exposure concentration below which there is no known risk to the health of presumptive “most vulnerable” (e.g., infants, children, pregnant women, elderly, immune-compromised) sub-populations (U.S. Environmental Protection Agency 2021d). Consistent with previous publications by this group, FDA SOQ (i.e., “shall not contain in excess of”) levels (U.S. Food & Drug Administration 2021) are presented to provide regulatory context; however, the EPA MCL values, on which FDA SOQ are generally based, take available treatment technologies and cost into consideration and, consequently, often are greater than corresponding human-health-only EPA MCLG values (U.S. Environmental Protection Agency 2021e).

### 3.1. BW exposure-benchmark comparisons – inorganics

No exceedances of FDA SOQ levels were observed for any inorganic analytes (Fig. 1, Table S3a). Few exceedances of human–health advisories for inorganics were observed in BW samples, with the notable exception of arsenic (As), uranium (U), and lead (Pb), which were broadly-detected here and widely reported at < MCL (less than the treatment technique action level for Pb) concentrations in previous BW studies in the US (e.g., Ikem et al. 2002; Saleh et al. 2008) and globally (e.g., Felipe-Sotelo et al. 2015; Krachler and Shotyk 2009) and which have no known safe level of exposure for vulnerable sub-populations (i. e., MCLG zero) (U.S. Environmental Protection Agency 2018).

Arsenic was not detected in any purified-TW BW (domestic) but was frequently detected (≥0.1 μg L^−1^) in domestic and imported spring-sourced BW (87 %), at concentrations up to greater than 7 μg L^−1^ in two domestic samples. Drinking-water As exposure is associated with various cancers (Mohammed Abdul et al. 2015; Smith and Steinmaus 2009), organ–system toxicity (Mohammed Abdul et al. 2015), cardiovascular disease (Navas-Acien et al. 2005; Pichler et al. 2019), diabetes (Navas-Acien et al. 2005; Pichler et al. 2019), adverse pregnancy outcomes (Shih et al. 2017), and mortality (Argos et al. 2010; Shih et al. 2017). Growing concerns for adverse health effects of drinking-water As concentrations less than the 10 μg L^−1^ EPA MCL (García-Esquinas et al. 2013; Mohammed Abdul et al. 2015; Navas-Acien et al. 2005; Navas-Acien et al. 2008) have prompted more strict public-supply MCL (e.g., 5 μg L^−1^ in New Hampshire and New Jersey) in some US states (New Hampshire Department of Environmental Services 2021; State of New Jersey 2021).

Likewise, U was frequently (74 %) detected (≥0.1 μg L^−1^) in domestic and imported spring–sourced BW, at concentrations up to 6.2 μg L^−1^ (imported) but was not detected in any purified–TW BW. Drinking-water U is associated with human nephrotoxicity (Magdo et al. 2007; Seldén et al. 2009) and osteotoxicity (Kurttio et al. 2005), DNA-repair inhibition in human embryonic kidney 293 (HEK293) cells (Cooper et al. 2016), and estrogen-receptor effects in mice (Raymond-Whish et al. 2007). Notably, As and U co-occurred in about 70 % (16) of spring–sourced BW samples in this study.

Pb was detected (≥0.1 μg L^−1^) in 5 of the 30 BW brands (17 %) at concentrations up to 1.1 μg L^−1^ and with comparable frequency in purified–TW and spring–sourced BW (14 % and 17 %, respectively). Public–health concerns for elevated drinking-water Pb–exposures are focused primarily on neurocognitive impairment in infants and children (Lanphear et al. 2016; Triantafyllidou and Edwards 2012), with the American Academy of Pediatrics recommending that drinking-water Pb not exceed 1 μg L^−1^ (Lanphear et al. 2016). Drinking-water Pb is attributed primarily to premise–plumbing and distribution–system infrastructures (Triantafyllidou and Edwards 2012) that predate the 1986 SDWA Amendments (U.S. Environmental Protection Agency 2021c).

Nitrate was routinely detected in BW samples in this study at concentrations generally consistent with previous BW comparison studies in the US (e.g., Ikem et al. 2002; Saleh et al. 2008) and globally (e.g., Felipe-Sotelo et al. 2015; Krachler and Shotyk 2009). Concentrations of NO_3_-N greater than 1 mg L^−1^ (including one at 8.1 mg L^−1^) were observed in 22 % (5/23) of spring–sourced BW samples in this study but not in any purified–TW BW. While the 10 mg L^−1^ MCLG was established to protect bottle-fed infants (<6 months) against methemoglobinemia (U.S. Environmental Protection Agency 2018, 2021e), drinking-water exposures to < MCLG NO_3_–N concentrations recently have been associated with several adverse outcomes (Ward et al. 2005; Ward et al. 2018), including cancers (Jones et al. 2016; Jones et al. 2017; Quist et al. 2018), thyroid disease (Aschebrook-Kilfoy et al. 2012), and neural tube defects (Brender et al. 2013).

Fluoride concentrations in all BW samples were below the 0.7 mg L^−1^ US Public Health Service drinking-water optimum to prevent childhood dental caries (2015), in line with previous concerns for the dental health of children, for whom BW is the primary drinking–water source (Cochrane et al. 2006; Horowitz et al. 2015; Mills et al. 2010). F concentrations were < 0.6 mg L^−1^ in all but one BW sample and < 0.3 mg L^−1^ in 28 (93 %) samples. F supplementation from 3 to 16 years of age is recommended for children with drinking-water F < 0.6 mg L^−1^, beginning at 6 months if F is < 0.3 mg L^–1^ (American Academy of Pediatrics: Committee on Nutrition 1995; Kohn et al. 2001).

### 3.2. BW exposure-benchmark comparisons - organics

Twenty-one (47 %) of the 45 organic analytes detected in this study were detected in 2 or fewer samples, with 14 (31 %) detected only once (Fig. 2, Table S4a). All but one BW sample (97 %) had at least one organic analyte detection, with more than one analyte detected in 87 % (26/45) of samples (Fig. 3 and Fig. 4). On average (median), 5 organics were detected per sample (interquartile range [IQR]: 2 – 6; range: 0 – 22), consistent with Hypothesis I, an anthropized water cycle, and previous private-/public-supply TW results by this research group (Bradley et al. 2018; Bradley et al. 2020; Bradley et al. 2021a; Bradley et al. 2021b; Bradley et al. 2022). In general, the most frequently detected organic analytes were DBP residuals of chlorine disinfection (e. g., trichloromethane, bromodichloromethane, acetonitrile, tribromo-methane), detected primarily in purified-TW BW but also in some spring-sourced BW (median: 4, IQR: 3 – 4 versus spring-sourced median: 0, IQR: 0 – 1), and a range of volatile organic chemical(s) (VOC) in both purified-TW (median: 2, IQR: 1 – 3) and spring–sourced (median: 2, IQR: 1 – 5) BW samples. Detection of trihalomethane DBP in purportedly untreated (i.e., no chlorine disinfection) spring-sourced BW samples has been reported previously (Stanhope et al. 2020). The total trihalomethane DBP concentration in one purified–TW BW sample was more than double the International Bottled Water Association’s code of practice limit of 10 μg L^−1^ (International Bottled Water Association 2020). In contrast to several previous studies (e.g., Akhbarizadeh et al. 2020; Chow et al. 2021; Gellrich et al. 2013; Luo et al. 2018; Wang et al. 2021), no PFAS, pharmaceutical, or phthalate contaminants were detected in this BW study. Likewise, pesticides were detected infrequently (4 samples), with the notable exception of one domestic, spring-sourced BW with 5 pesticide detections (cumulative concentration 0.119 μg L^−1^).

In line with Hypothesis II, no exceedances of FDA SOQ levels were observed for organic analytes in this study. Fifteen of the 45 organics detected in this study (33 %) have EPA promulgated MCLG. Among these, 3 DBP (bromodichloromethane [8 samples], tribromomethane [5], dichloromethane [4]) and 3 VOC (tetrachloroethene [5], benzene [3], trichloroethene [1]) have no known safe level of exposure for vulnerable sub-populations (i.e., MCLG of zero) (U.S. Environmental Protection Agency 2018), representing de facto exceedances (Hypothesis III). Simultaneous exposures to multiple organic contaminants in these samples expand the concern-space for potential biological effects of POU drinking water exposures to include BW, emphasizing the need for improved understanding of the adverse human-health implications, if any, of long-term exposures to low–level organic-contaminant mixtures across all 3 drinking-water distribution pipelines (private–, public–, and bottled–supply).

### 3.3. BW exposure-benchmark comparisons - microbial

Viable bacteria were detected by heterotrophic plate count (HPC) or by microscopic direct counts in 97 % (29/30) of BW samples (6/7 purified-TW, 23/23 spring–sourced) and at concentrations greater than 100 HPC CFU 100 mL^−1^ in 17 % of samples, all spring-sourced (Table S5). HPC bacteria occur naturally in the environment, are commonly detected in drinking water, and are not intrinsic health concerns but are useful indicators of source-water quality, system maintenance, disinfection efficacy, and post-treatment regrowth in the distribution “pipeline” prior to consumption (U.S. Environmental Protection Agency 2021e). To optimize detection of viable heterotrophs, BW samples were assessed with two growth media (SimPlate, R2A) and incubation durations (2 and 4 days). In general, detections of heterotrophs by HPC and of bacteria and virus-like particles by direct microscopic counts were more common in spring–sourced BW than in purified-TW BW; the latter were all derived from chlorine-disinfected TW (as indicated by presence of chlorine DBP) and treated by reverse-osmosis advanced filtration (according to label). Reduction and avoidance of chlorine-disinfection DBP and associated tastes/odors are common considerations, respectively, for advanced filtration (e.g., reverse osmosis) of purified-TW BW and for use of non-chlorine, advanced–oxidation (ozonation, ozonation/UV radiation) for spring–sourced BW disinfection, when employed. While two of the highest HPC results were observed in spring-sourced BW with no listed filtration or treatment (BW03, BW29), comparable high results for ozone–/UV–disinfected BW illustrate the trade-off of reduced DBP/taste concerns but increased biological regrowth concerns in the absence of residual disinfectant. Growth on putative pathogen selective media was observed sporadically across all BW samples, albeit at near detection-limit levels.

### 3.4. BW in vitro bioactivities

Given the potential low estrogenic activity in the sample extracts, a tiered screening approach was applied to sample analysis as previously described (Medlock Kakaley et al. 2021). No BW sample extract produced estrogenic activity significantly greater than control treated cells (p < 0.01) and, therefore, none exceeded the bioassay minimum detectable concentration (MDC; 0.057 ng/L) for estrogenic activity. Estrogenic activity, generally below estimated trigger values for adverse effects (Neale and Escher 2019), has been reported previously in treated TW in the US (Conley et al. 2017b) and globally (Brand et al. 2013; Maggioni et al. 2013; Van Zijl et al. 2017) and in BW (Aneck-Hahn et al. 2018; Real et al. 2015; Wagner and Oehlmann 2009).

### 3.5. BW aggregated screening assessments

Potential human-health effects of BW contaminant–mixture exposures were screened using cumulative bioactivity-weighted approaches based on detected analytes. The Σ_EAR_ and Σ_TQ_ approaches employed herein and in the previous TW studies 1) are constrained intrinsically by the analytical scope, which, although extensive (in this case 465 unique organics and 53 inorganics), remains orders-of-magnitude below estimates of anthropogenic chemicals in commercial production (Wang et al. 2020) and, by extension, potentially present in ambient drinking-water source waters (Bradley et al., 2017; de Jesus Gaffney et al. 2015; DeSimone et al. 2015; Toccalino et al. 2012), 2) are limited by available weighting–factors (ToxCast ACC and human health benchmarks, respectively), and 3) estimate mixture effects assuming approximate concentration addition (e.g., Cedergreen et al. 2008; Ermler et al. 2011; Stalter et al. 2020). The Σ_EAR_ approach (Blackwell et al. 2017; Bradley et al. 2021a) employs ToxCast high-throughput exposure-effects data to predict potential cumulative bioactivity at the molecular scale (Filer et al. 2017; Richard et al. 2016); however, ToxCast has no coverage of inorganic contaminants and not all predicted organic-contaminant molecular responses are necessarily adverse at organ/organism scales (Schroeder et al. 2016). We aggregated contaminant bioactivity ratios across all ToxCast endpoints without restriction to recognized modes of action to provide a precautionary lower-bound estimate of *in vivo* adverse-effect levels (Paul Friedman et al. 2020), but this approach may not accurately reflect apical effects (Blackwell et al. 2017; Schroeder et al. 2016). In contrast, the employed Σ_TQ_ approach targets apical human-health effects, includes inorganic exposures, but is notably constrained to recognized (i.e., benchmarked) health concerns. Importantly, the EAR approach is based on measured endpoint-specific activity cutoff concentrations, whereas the human-health benchmarks used in the TQ approach include a margin of safety (margin of exposure).

**Fig. 5.**
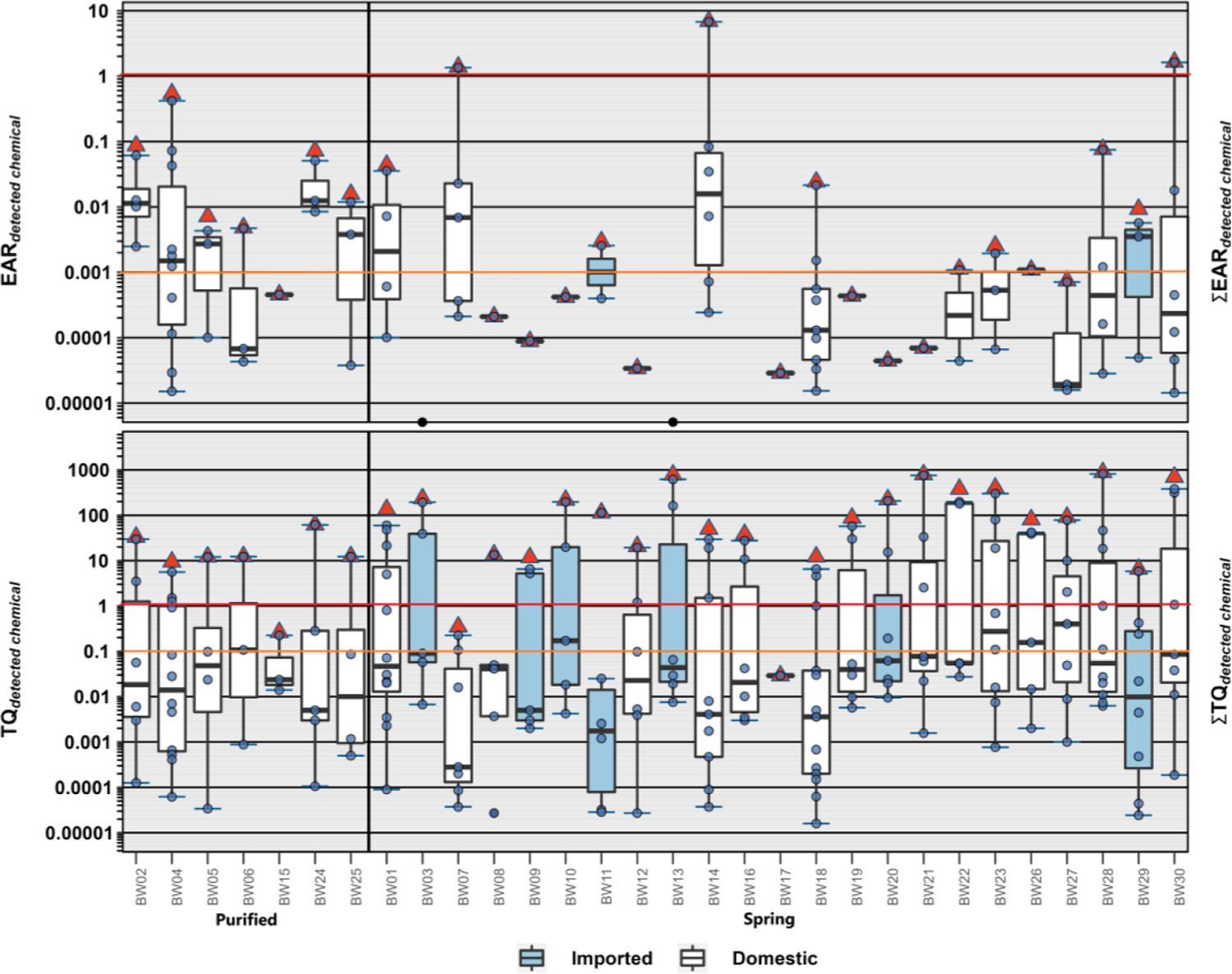
**Top.** Individual EAR values (circles) and cumulative EAR (Σ_EAR_, sum of all detected; red triangles, △) across all assays for 24 organic analytes listed in ToxCast and detected in spring (domestic, white; imported, blue) and purified-TW (domestic) sourced bottled water samples during 2020. Solid and dashed red lines indicate concentrations shown to modulate effects *in vitro* and effects-screening-level thresholds (EAR = 0.001), respectively. Circles on x-axis (BW03 and BW13) indicate EAR < 0.00001. **Bottom.** Human health benchmark-based individual TQ values (circles) and cumulative TQ (Σ_TQ_, sum of all detected; red triangles, △) for inorganic and organic analytes listed in Table S7a and detected in spring (domestic, white; imported, blue) and purified-TW (domestic) sourced bottled water samples during 2020. Solid and dashed red lines indicate benchmark equivalent concentrations and effects-screening-level threshold of concern (TQ = 0.1), respectively. Boxes, centerlines, and whiskers indicate interquartile range, median, and 5th and 95th percentiles, respectively, for both plots.

Twenty-six of the 45 organic contaminants detected in BW samples had exact Chemical Abstract Services (CAS) number matches in the ToxCast invitroDBv3.2 database (Figure S2, Table S6b). The highest individual EAR values (1.35–6.74) and the only EAR and Σ_EAR_ exceeding the level expected to modulate molecular targets *in vitro* (i.e., solid red Σ_EAR_ = 1 line, Fig. 5) in this study were for three spring-sourced BW samples containing μg L^−1^ concentrations of the VOC, 1–butanol. Acknowledging the incomplete (58 %) ToxCast coverage of the detected organic analytes, the potential 2–3 orders–of–magnitude analytical underestimation of the presumptive exposure space (465 unique analytes compared to estimated 350,000 commercial organic compounds and presumptive greater number (Dobson 2004) of corresponding degradates and metabolites in the environment), the recognized vulnerability of specific populations (Blake and Fenton 2020), and the reported approximate contaminant-specific equivalency to the widely employed TQ = 0.1 screening-level threshold for low risk (Corsi et al. 2019), a precautionary Σ_EAR_ = 0.001 screening–level was employed, as described. Exceedances of Σ_EAR_ = 0.001 in more than half (17/30) of the BW samples were attributable primarily to DBP in purified-TW BW and to a variety of VOC, including trihalomethanes, in spring-sourced BW and were most consistently associated with DNA-binding endpoints (Table 6c); based on these results, further investigation of the cumulative molecular activity of low-level BW chemical exposures is warranted.

All but one of the BW samples in this study exceeded the Σ_TQ_ = 0.1 screening threshold of potential concern, with most (27/30) exceeding Σ_TQ_ = 1 (Figures 5, S3; Table S7b). These Σ_TQ_ results indicate high probabilities of cumulative risk in the tested BW samples, when considering both organic and inorganic contaminant exposures. Exceedances of Σ_TQ_ = 1 were driven primarily by DBP in purified-TW BW samples, all of which were labeled as RO treated; incomplete or poor RO rejection of low molecular weight organics including trihalomethane DBP is well-documented (Breitner et al. 2019; Marron et al. 2019). Exceedances of Σ_TQ_ = 1 in spring–sourced BW samples also were primarily attributable to trihalomethane compounds, with tribromomethane and bromodichloromethane, compounds with no known safe level of exposure (MCLG = 0), alone exceeding the threshold in 5 (22 %) and 2 (8 %) spring-sourced BW samples, respectively. Other notable Σ_TQ_ results included frequent, often co-occurring, detections of As, Pb, and U, as noted above. These results indicate that simultaneous exposures to multiple drinking-water contaminants of potential human-health concern are common in BW, emphasizing the need for improved understanding of the adverse human-health implications, if any, of long-term exposures to low–level inorganic-/organic-contaminant mixtures across all three drinking-water distribution pipelines (public TW, private TW, BW).

### 3.6. Study limitations and future research

For this initial reconnaissance of the potential for human exposures to an expanded range of inorganic, organic, and microbial contaminants in BW, 30 BW brands were selected to broadly represent the range of source locations, source waters, pre-distribution treatments, and packaging materials of commercially available BW across the US. However, BW is the largest commercial beverage category by volume in the US and the 30 brands assessed herein are a small fraction of those available in the US and globally (Rodwan 2021); further latitudinal (more brands) and longitudinal (temporal variability) assessment is required to fully inform the range of BW exposures in the US and globally. Likewise, as noted above, the target analytical scope of this and the previous POU–TW studies (Bradley et al. 2018; Bradley et al. 2020; Bradley et al. 2021a; Bradley et al. 2021b; Bradley et al. 2022), while extensive and environmentally relevant, is only a fractional indicator of anthropogenic chemicals in commercial production and potentially present in ambient drinking-water source waters; accordingly, the exposure and associated risk results may be reasonably interpreted as potential orders-of-magnitude underestimates. Other inherent limitations of the EAR and TQ risk assessment approaches are discussed in the previous section. Additionally, the EAR risk assessment approach herein and in the previous POU–TW studies (Bradley et al. 2018; Bradley et al. 2020; Bradley et al. 2021a; Bradley et al. 2021b; Bradley et al. 2022) employs measured BW concentrations as a direct estimate of human exposure. Alternatively, the ToxCast molecular–endpoint ACC data may be viewed as more aligned with internal doses and the EAR risk assessment conducted using internal doses estimated by, for example, high-throughput toxicokinetic modeling using the *httk* R package (Pearce et al. 2017), as described (Paul Friedman et al. 2020). Lastly, the ToxCast datasets and to a more limited extent the benchmarks employed in the Σ_EAR_ and Σ_TQ_ risk assessments, respectively, have been routinely updated; accordingly, in contrast to the exposure results, direct comparison of estimated risks between this and the previous POU–TW studies requires harmonization of the ACC and benchmark data and was beyond the scope of the current study. Important next steps include harmonization of toxicity-weighting data and direct comparison of the Σ_EAR_ and Σ_TQ_ risk assessments based on POU–DW exposure data, comparative assessment of EAR risk based on the internal dose estimation approach of Paul Friedman et al. 2020, continued target assessment of broad inorganic, organic, microbial exposures and associated risks across all three POU-DW distribution pipelines, and incorporation of non-targeted chemical and effects-based assay platforms to more broadly characterize human POU-DW exposures and risks.

## 4. Conclusions

In the US and globally, drinking water is delivered to consumers via three distribution systems (public TW, private TW, BW), each having distinct logistical, infrastructure, regulatory, and commercial profiles, but all similarly challenged by anthropogenic water-quality concerns. Attributed largely to commercial promotion as a safer alternative to public and private TW (Hawkins 2017; Olson et al. 1999), BW consumption has increased dramatically in the US (Rodwan 2021) amidst heightened anxieties about environmental contaminant exposures and health risks (Pape and Seo 2015; Zivin et al. 2011), despite a paucity of directly comparable, realistically-broad contaminant exposure data for BW. This data gap impedes individual-consumer risk–management decision making.

In this study, 48 inorganics and 45 organics, including some with documented human-health concerns, were detected in sampled BW. While no FDA SOQ levels were exceeded in any BW samples, several inorganic and organic contaminants with MCLG of zero (no known safe level of exposure to vulnerable sub-populations) were detected, in some cases at near–MCL concentrations. Among these, As, Pb, and U were detected in 67 %, 17 %, and 57 % of BW samples, respectively, and almost exclusively in spring-sourced BW samples, which were not treated by the advanced filtration (e.g., reverse osmosis) methods applied to all the purified–TW sourced BW samples assessed herein. Organic MCLG exceedances included frequent detections of DBP in TW–sourced BW and infrequent detections of VOC in purified–tapwater and spring–sourced BW. Precautionary health–based HI screening levels were exceeded frequently and attributed primarily to chlorine–disinfection DBP in purified-tapwater sourced BW and to co-occurring inorganic (e.g., As, U) and organic (e.g., brominated trihalomethanes) contaminants in spring-sourced BW. While extensive (465 unique organics, 53 inorganics), the organic analytes assessed in this study are an orders–of–magnitude underestimate of the breadth of anthropogenic chemicals in commercial production (Wang et al. 2020) and, thus, presumptive fractional indicators of potential BW exposures and risk. The results of this one-time reconnaissance of a limited number of BW brands (30) indicate that simultaneous exposures to multiple drinking-water contaminants of potential human-health concern are common in BW and illustrate the need for further directly–comparable, realistically-broad contaminant exposure assessments across a broader range of BW brands and over time.

**Fig. 6.**
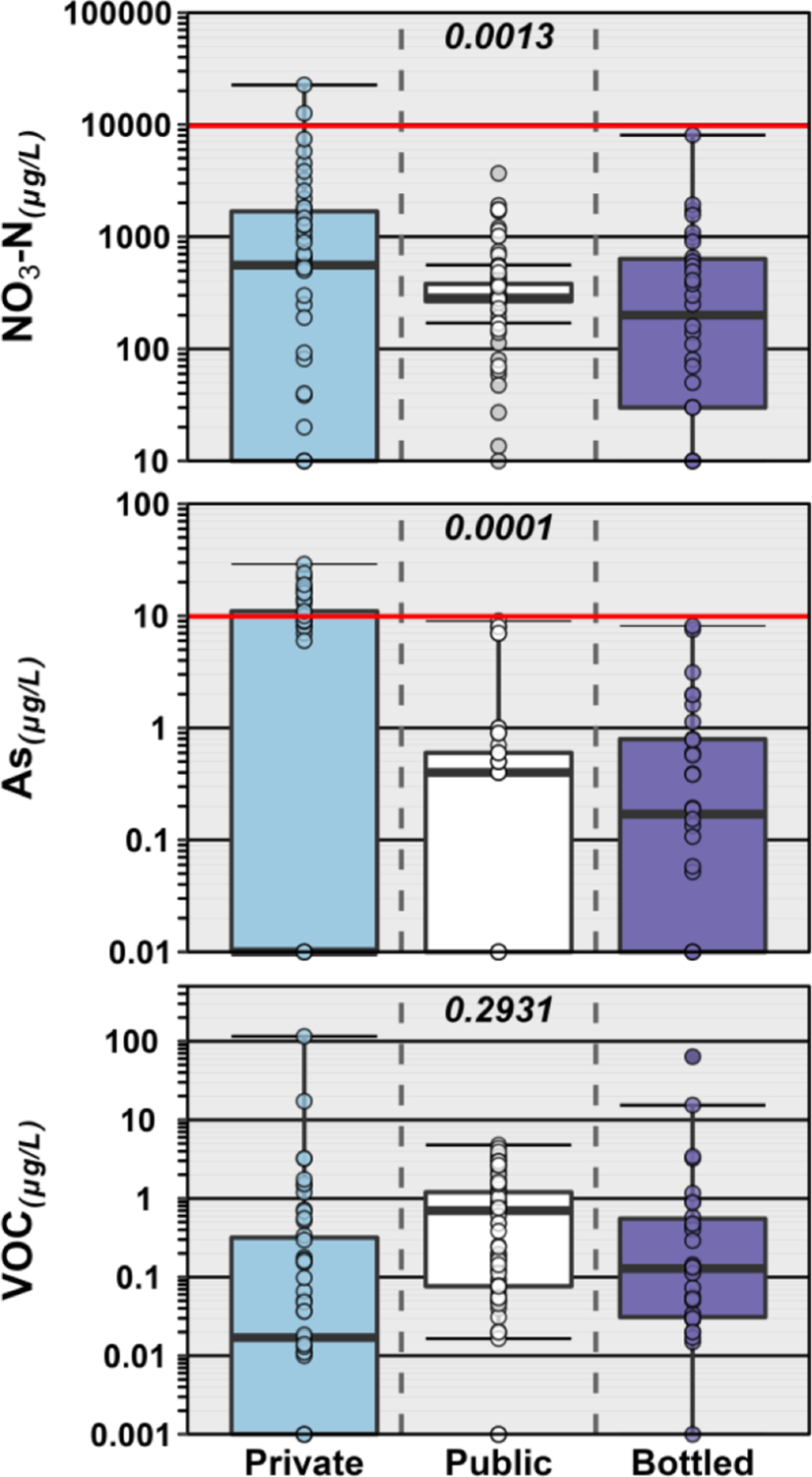
Group comparison of concentrations of NO_3_-N (top), As (middle), and VOC (bottom) detected in private TW (blue) and public TW (white) in previously published studies and in BW (purple) herein. Solid red lines indicate enforceable FDA SOQ levels. For NO_3_-N, SOQ and MCLG are the same. MCLG for As is zero. Boxes, centerlines, and whiskers indicate interquartile range, median, and 5th and 95th percentiles, respectively. Numbers (top center of plots) indicate the permuted probability that the centroids and dispersions are the same (PERMANOVA; 9999 permutations) across all groups.

Importantly, comparison of these BW exposure results with those of the previous POU–TW studies documents the shared challenge to all three POU-DW distribution pipelines posed by the increasingly anthropized water cycle. To illustrate, Fig. 6 displays sample results for select major ion (NO3-N), trace metal (As), and organic (VOC) contaminants detected in this BW study and in the previous POU–TW studies (Bradley et al. 2018; Bradley et al. 2020; Bradley et al. 2021a; Bradley et al. 2021b; Bradley et al. 2022). Considerable variability in POU-DW contaminant exposures exists for private, public, and bottled DW supplies, with generally comparable ranges in contaminant concentrations observed to date across all three distribution pipelines. The results to date do not support market-driven perceptions of BW as systematically higher purity than public TW and emphasize the need for improved source-water protection, monitoring and characterization, and treatment options across all three pipelines.

Improved understandings of point-of-use drinking-water contaminant exposures based on more environmentally realistic and directly comparable POU-exposure characterizations, like this BW study and previous TW studies by this group (Bradley et al. 2018; Bradley et al. 2020; Bradley et al. 2021a; Bradley et al. 2021b; Bradley et al. 2022), are essential to public health, because drinking-water is a biological necessity and, consequently, a high-vulnerability vector for human contaminant exposures (Dai et al. 2017). The results illustrate the importance of continued systematic, quantitative assessments of realistically-broad contaminant exposures and associated bioactivities in POU drinking water from all three distribution pipelines (private TW, public TW, and BW) to support models of drinking-water contaminant exposures and related risks at the point of use.

## Supplementary Material

Supplement1

## Data Availability

All data are available in the supporting information file and in the cited Romanok et al. 2022 USGS Data Release.
